# Chelators for copper radionuclides in positron emission tomography radiopharmaceuticals^†^

**DOI:** 10.1002/jlcr.3165

**Published:** 2013-12-18

**Authors:** Zhengxin Cai, Carolyn J. Anderson

**Affiliations:** Department of Radiology, University of Pittsburgh, Pittsburgh, PA 15219, USA

**Keywords:** PET, copper 64, chelator, molecular imaging, radiopharmaceutical

## Abstract

The development of chelating agents for copper radionuclides in positron emission tomography radiopharmaceuticals has been a highly active and important area of study in recent years. The rapid evolution of chelators has resulted in highly specific copper chelators that can be readily conjugated to biomolecules and efficiently radiolabeled to form stable complexes *in vivo*. Chelators are not only designed for conjugation to monovalent biomolecules but also for incorporation into multivalent targeting ligands such as theranostic nanoparticles. These advancements have strengthened the role of copper radionuclides in the fields of nuclear medicine and molecular imaging. This review emphasizes developments of new copper chelators that have most greatly advanced the field of copper-based radiopharmaceuticals over the past 5 years.

## Introduction

Positron emission tomography (PET) is one of the molecular imaging modalities that probes physiological changes noninvasively, and its hybridization with anatomical imaging modalities, such as computed tomography (CT) and magnetic resonance imaging have made great contributions to the diagnosis of disease over the past few decades.^1^ The success of PET imaging relies on a synergistic effort of biomarker identification, radiopharmaceutical discovery, and development and instrument innovation. Along with [^18^F]FDG, which images glucose metabolism, there are a plethora of probes for imaging other biological processes and biomarkers, although only a handful are approved for human use.^2^

Because of the short half-lives of the traditional positron-emitting radionuclides, such as ^18^F and ^11^C, radionuclides with longer half-lives are needed for monitoring *in vivo* processes with longer biological half-lives using longer circulating antibodies and nanoparticles. Radiometals such as ^64^Cu (T_1/2_ = 12.7 h), ^86^Y (T_1/2_ = 14.74 h), and ^89^Zr (T_1/2_ = 78.4 h), fall into this category.

The development of bifunctional chelators plays a pivotal role in metal-based radiopharmaceuticals.^3,4^ Copper has unique chemical characteristics that make the use of its radiometals particularly interesting.^5^ Copper has many important biological roles *in vivo*, such as electron transfer, catalysis, and structural shaping.^6^ Many copper-containing compounds are biologically active and have anti-inflammatory and antiproliferative properties.^7,8^ Copper radionuclides, such as ^60^Cu, ^61^Cu, ^62^Cu, ^64^Cu, and ^67^Cu (Table 1) offer versatile choices for applications in imaging,^9^ therapy,^10,11^ or theranostics. Copper-64, with a half-life of 12.7 h, and its unique decay profile (β^+^: 18%; β^−^: 38%; electron capture: 44%),^12^ is well suited for radiolabeling nanoparticles, antibodies, antibody fragments, peptides, or small molecules for PET imaging, radio-therapy, and drug discovery and development.^13^ Copper-64 chloride is produced using a biomedical cyclotron in high specific activity and is commercially available.^14,15^

In the development of Cu-64 radiopharmaceuticals, chelator design is essential, because it is required for complex Cu(II), and for many imaging probes, to attach it to biomolecules and generate kinetically and thermodynamically stable systems. The development of copper chelators is especially challenging as there are many copper chelating proteins *in vivo* (e.g., ceruloplasmin, superoxide dismutase, metallothionein, copper transporters, and chaperones) that potentially can displace the copper ion from the chelator.^16^ Of the different oxidation states of copper, Cu(II) is most common for Cu-64 radiopharmaceuticals. With a 3d^9^ electronic configuration, Cu(II) has borderline hardness, favoring binding to nitrogen donors. The coordination number ranges from four to six, forming complexes that have square planar, square pyramidal, trigonal bipyramidal, or octahedral geometry. Jahn–Teller distortion occurs and this is observable by the X-ray crystallography in the molecular structures of Cu(II) complexes.

**Figure 1:**
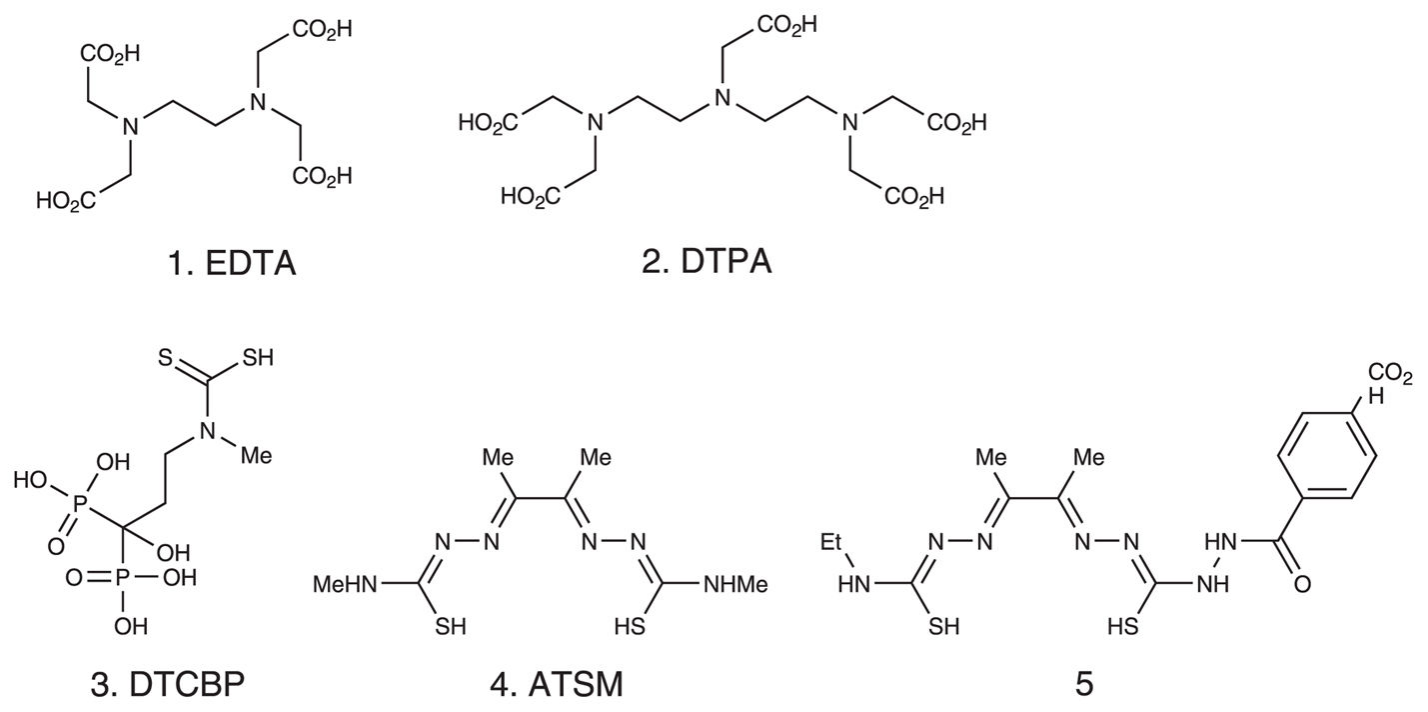
Examples of acyclic chelators.

**Figure 2:**
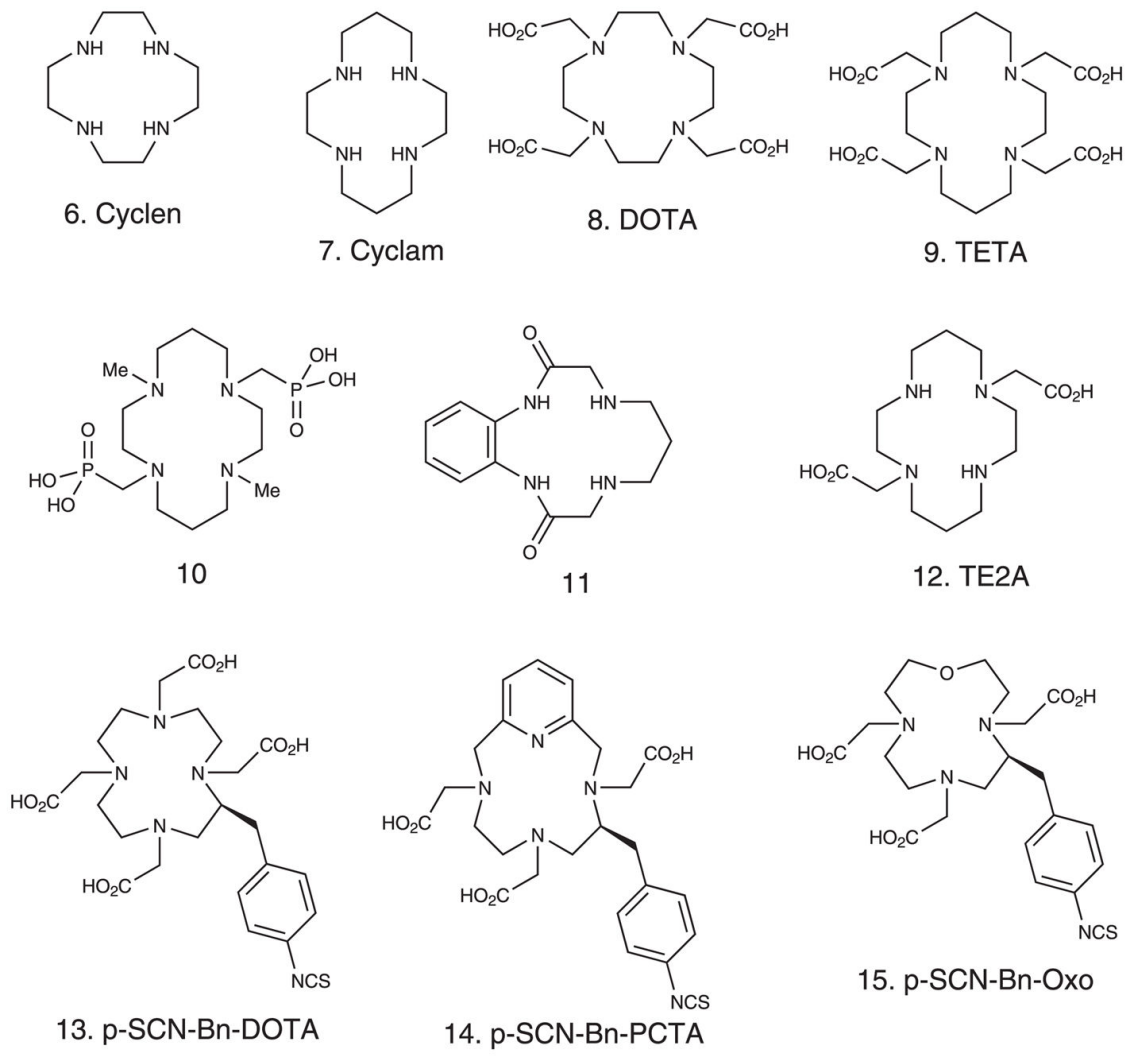
Examples of nonbridged macrocyclic chelators.

**Figure 3:**
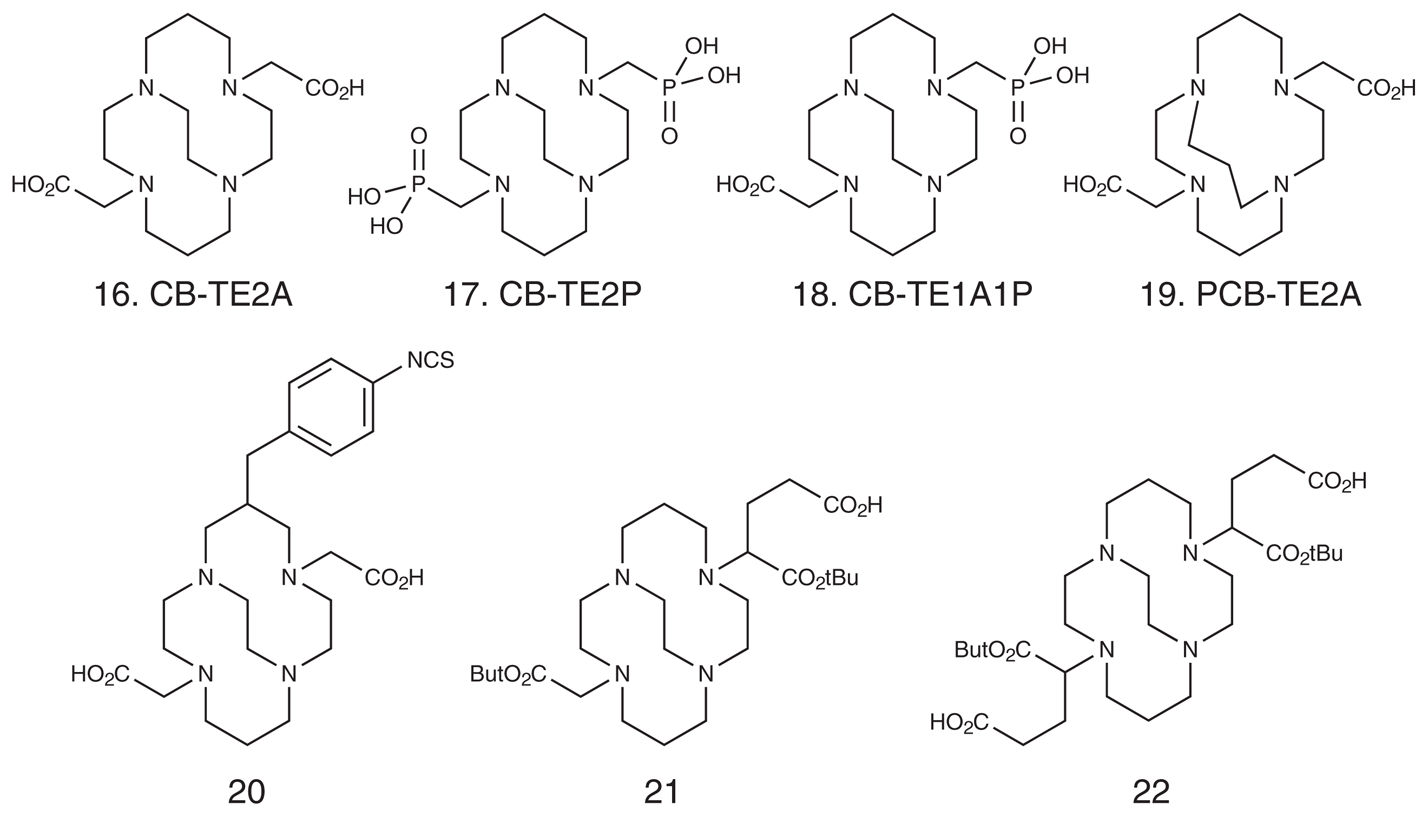
Examples of cross-bridged macrocyclic chelators.

**Figure 4:**
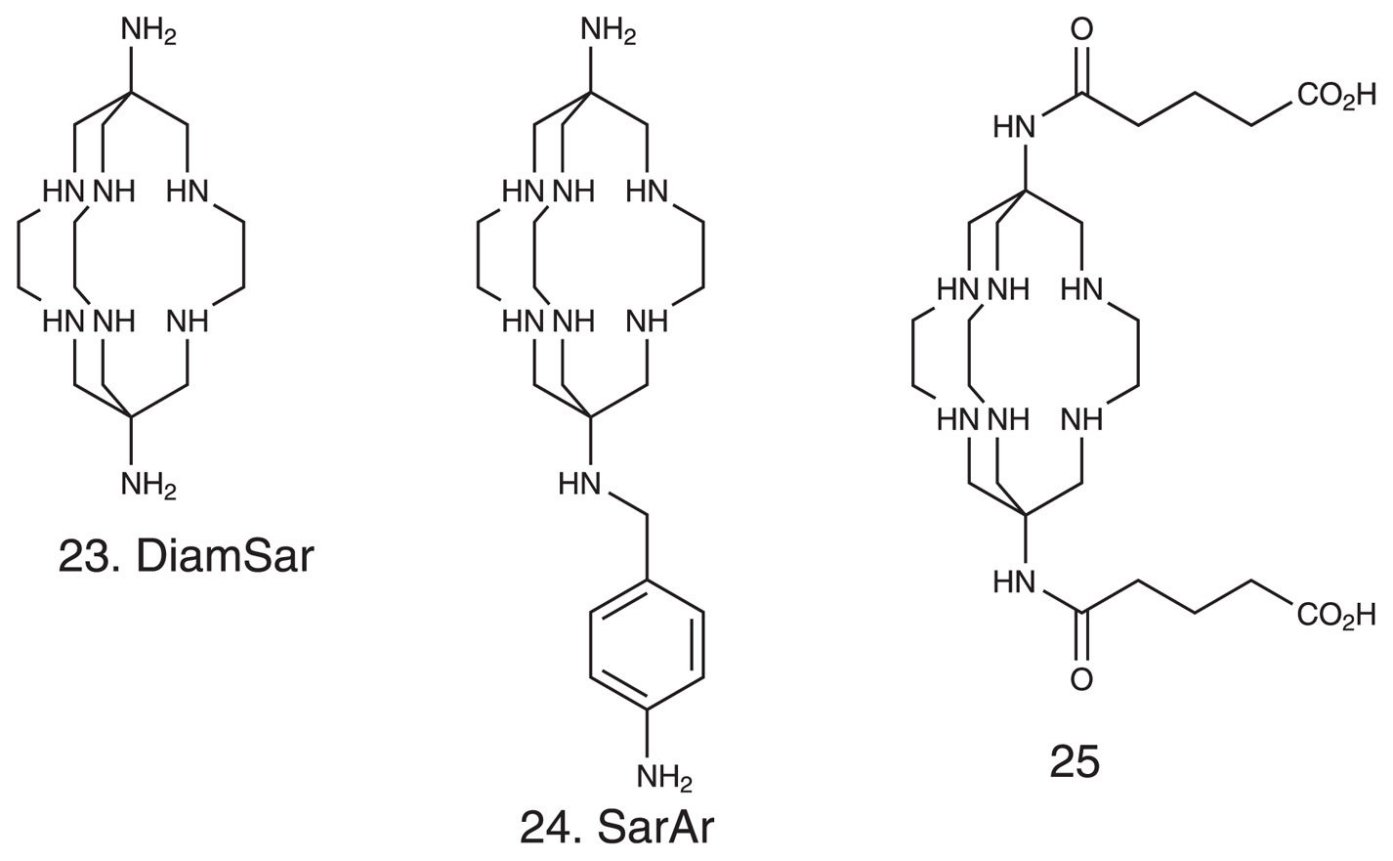
Examples of sarcophagine-based chelators.

**Figure 5:**
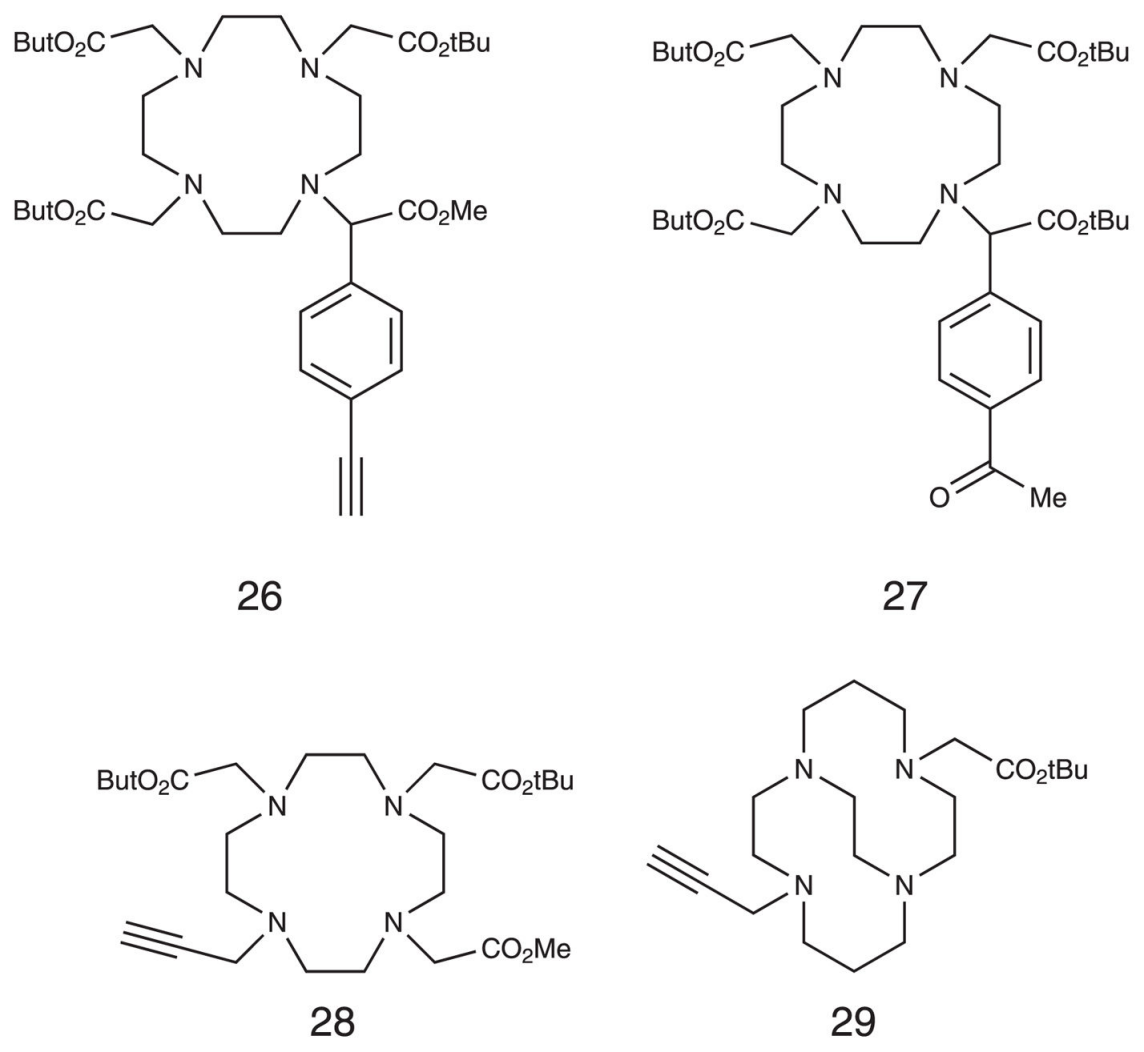
Examples of clickable chelators.

**Figure 6:**
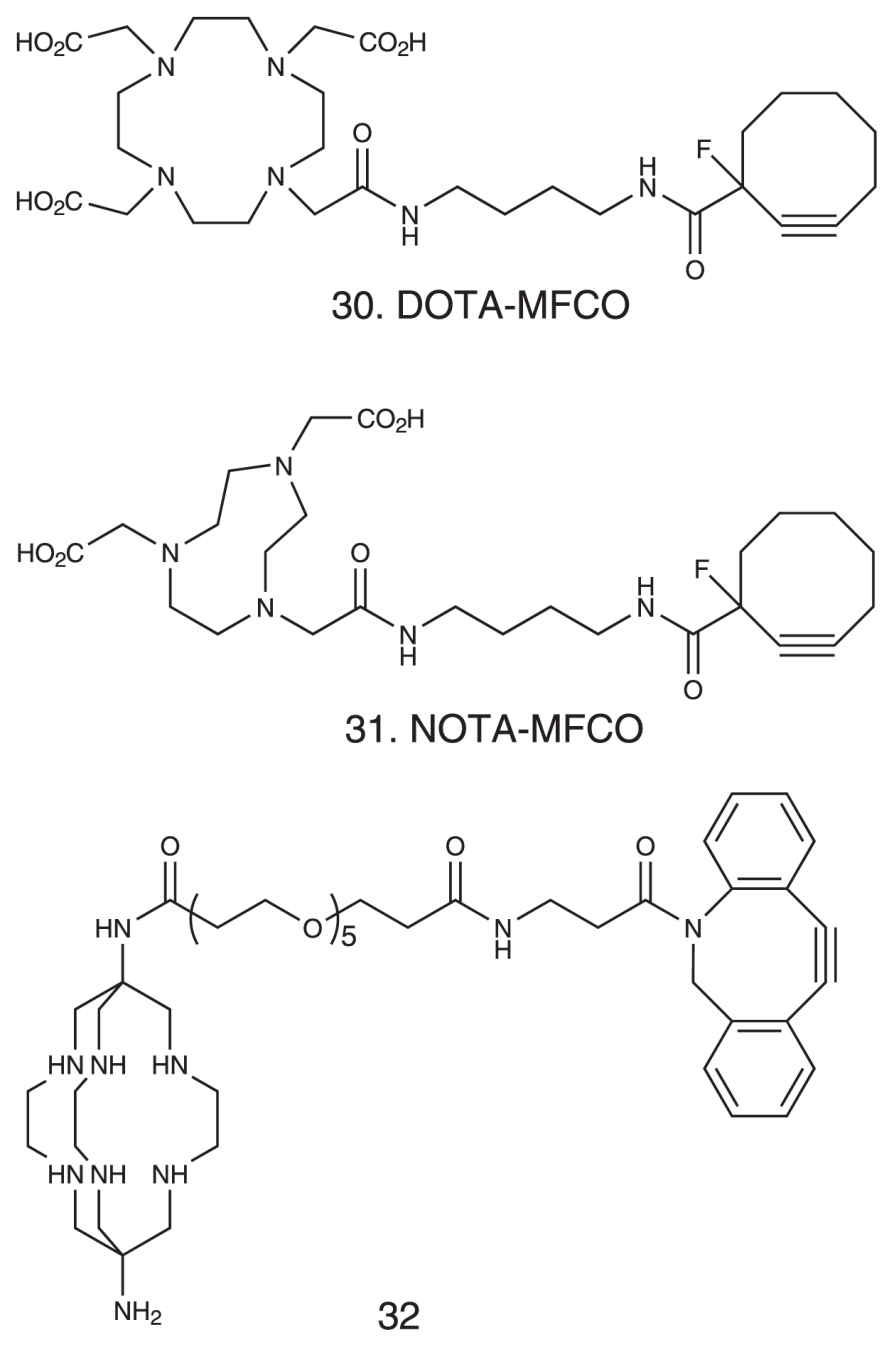
Examples of strain-promoted clickable chelators.

This mini review will focus on recent developments of copper chelators for use in PET radiopharmaceuticals (primarily in the past 5 years).^17–19^ It includes innovations in chelators with novel structures and modification of known chelators with functional groups amenable for bioconjugation (Figures 1–6). Methods for *in vitro* evaluation of copper chelate stability, nonchelating nanocarriers of copper-64, and factors involved in the specific activity of copper radionuclides are also discussed.

### Acyclic chelators

Classical acyclic copper chelators include ethylenediaminetetraacetic acid (**1**. EDTA), diethylene triamine pentaacetic acid (**2**. DTPA), and their derivatives. Dithiocarbamates have been used for chelating copper, although these are typically used for chelating technetium and rhenium in nuclear medicine.^20^ Torres Martin de Rosales *et al*. reported a dual-modality PET–MRI agent based on ^64^Cu-dithiocarbamatebisphosphonate (**3**. DTCBP conjugated to iron oxide nanoparticles.^21^ This was successfully used to image draining lymph nodes in a mouse model. Although the copper complex with DTCBP is not highly kinetically stable, with a half-life of less than 5 min in 5 M HCl at 90°C compared with 154 h for Cu-CB-TE2A, the *in vivo* imaging showed retention of the probe in popliteal and iliac lymph nodes.^22^ Bis(thiosemicarbazones) such as diacetyl-bis(N4-methylthiosemicarbazone) (**4**. H_2_ATSM) were developed as copper chelator mainly for hypoxia imaging and have been previously reviewed extensively.^23,24^ The application of bis(thiosemicarbazones) as bifunctional chelators is rare. However, Hueting *et al*. reported their evaluation of them, such as **5**, using a bombesin derivative as the model peptide.^25^

### Nonbridged macrocyclic chelators

Because of the macrocyclic effect, macrocyclic chelators generally form kinetically more stable Cu(II) complexes than acyclic chelators. The classical macrocycles used to chelate Cu (II) for PET imaging are cyclen (**6**) and cyclam (**7**) modified with N-acetic acid arms, with the most commonly used being DOTA (**8**) and 1,4,8,11-tetraazacyclododecane-1,4,8,11-tetraacetic acid (TETA) (**9**).^26–28^ However, *in vivo* instability of these complexes has been demonstrated.^29^ Svobodová, *et al*. efficiently synthesized a dimethyl-diphosphonate cyclam derivative **10** that has square-pyramidal or trigonal-bipyramidal geometry with copper depending on the extent of protonation of the chelator, leaving one phosphoric acid arm that is not chelated with Cu(II).^30^ This complex is kinetically labile, losing the copper quickly under acidic conditions. Barnard, *et al*. made a diamide macrocyclic chelator (**11**) and showed that the proton on the nitrogen of the amide was deprotonated, forming a distorted square planar complex with Cu(II). A biodistribution study with the ^64^Cu-labeled compound showed rapid blood clearance but with much higher liver uptake and retention than CB-TE2A, indicating its instability *in vivo*.^31^ Pandya *et al*. reported a synthesis of TE2A (**12**), and the acid decomplexation and reduction potential measurements indicated that Cu-TE2A is more stable than Cu-TETA, on the basis of rat biodistribution data showing significantly lower retention of ^64^Cu-TE2A than ^64^Cu-TETA in kidney and liver at 24 h post injection, suggesting a lower extent of demetallation *in vivo*.^32^ In a follow-up paper, a new synthesis of the salt-free TE2A was reported that was conjugated to c(RGDyK), but no biodistribution data were reported.^33^ Zhang *et al*. reported a multivalency platform based on the DOTA chelator using c(RGDyK) with no space between the c and (RGDyK) as a model peptide.^34^ Interestingly, the dimer outperformed the corresponding monomer and trimer as a PET-imaging agent. Ferreira *et al*. compared *p*-SCN-Bn-DOTA (**13**) with two newly developed chelators, *p*-SCN-Bn-PCTA (**14**) and *p*-SCN-Bn-Oxo (**15**) conjugated with trastuzumab.^35^ Both **14**-trastuzumab and **15**-trastuzumab were superior to the DOTA analogue, with respect to labeling efficiency, serum stability, and tumor uptake. Subsequently, Cooper *et al*. reported a systematic comparison of eight chelators including **13, 14**, and **15**, after conjugation with antibody rituximab.^36^ They were able to label the DOTA conjugate at low temperatures in 20 min, and the labeled conjugate was stable in serum for up to 48 h. They speculated that the discrepancy with other reports on DOTA-based antibody conjugates came from the source of Cu-64 they used, emphasizing the importance of the radionuclide purity, especially for less copper-selective chelators such as DOTA.

### Cross-bridged macrocyclic chelators

Because the *in vivo* stability of Cu(II) complexes is vital for PET imaging, the incorporation of 1,8-ethylene cross-bridged macrocyclic chelators into ^64^Cu radiopharmaceuticals was an important advance. Among several cyclen and cyclam-based cross-bridged macrocyclic chelators, the lead chelator was CB-TE2A (**16**), which showed improved *in vivo* stability over DOTA and TETA.^37–39^ However, because of the requirement of harsh radiolabeling conditions (95°C for 60 min), the application of this chelator is limited to heat insensitive molecules, precluding its application in antibodies or other proteins.^40^ It is noteworthy that Yoo and colleagues developed an efficient synthesis of propylene cross-bridged TE2A (**19**).^41^

Compound **19** showed high *in vitro* and *in vivo* stability and can be labeled under milder conditions than the corresponding ethylene cross-bridged **16**. A large series of propylene cross-bridged tetraaza macrocyclic compounds were patented by the same authors.^42^

To keep the two pendant arms of CB-TE2A available for chelating copper and neutralizing the positive charge when one of the carboxylates is utilized for conjugation to a biomolecule, Lewis *et al*. introduced a 4-nitrobenzyl group at the 6-position of the cyclam backbone, which was converted to an amine-reactive isothiocyanate (**20**) for easy conjugation to biomolecules.^43^ The radiolabeling and stability studies of the radio-Cu(II) chelate have not been reported to our knowledge. Boswell *et al*. functionalized cross-bridged cyclam with orthogonally protected 2-bromopentanedioic acid to get an asymmetrically substituted cross-bridged macrocyclic chelator (**21**).^44^ However, the labeling conditions were similar to those used for CB-TE2A, that is, requiring high temperatures. Liu *et al*. went further to generate the bis-pentanedioic acid modified cross-bridged cyclam (**22**).^45^ Using c(RGDyK) as a model peptide ligand, they tested the concept of multivalency on the basis of this platform. The results showed that the bivalent probe was superior in uptake and retention in tumors than the corresponding monomer.

Additional efforts to find a chelator that could be labeled at lower temperatures resulted in the phosphonate-armed chelators, such as CB-TE2P (**17**) and CB-TE1A1P (**18**), which can be labeled at room temperature within 1 h in high specific activity.^46,47^ The pharmacokinetics of these Cu(II) phosphonate CB complexes were compared with copper complexes of CB-TE2A, diamsar, and NOTA, and exhibited rapid blood clearance with minimum retention in liver, kidney, and bone marrow.^46^ This is in contrast with the noncross-bridged analogues, such as **10** published by Svobodová, *et al*.^30^ One of the phosphonate cross-bridged chelators, CB-TE1A1P, was conjugated to the somatostatin analog, Y3-TATE,^48^ and the α_4_β_1_ integrin-targeting agent, LLP2A.^49 64^Cu-CB-TE1A1P-Y3-TATE could be labeled in ~90% purity within 1 h at 40°C, whereas ^64^Cu-CB-TE1A1P-LLP2A was 100% labeled by 1 h at room temperature, likely because of a longer linker between the chelator and the biomolecule. The biodistributions of both compounds were improved over CB-TE2A analogs, having greater tumor uptake and improved contrast.^48,49^

### Sarcophagine-based chelators

Sarcophagine chelators were pioneered by Sargeson and colleagues in the late 1970s.^50^ A comparison of CB-TE2A and diamsar (**23**) conjugated RGD peptides was performed, and the data suggested that a linker between the diamsar chelator and the peptide would improve the targeting and biodistribution.^51^ SarAr (**24**) includes the diamsar chelator as well as a linker, allowing for improved labeling efficiency after conjugation.^52^ Ma *et al*. functionalized both of the primary amines with succinic or glutaric anhydride (**25**) and conjugated the chelators with Tyr^3^-octreotate or with two equivalents of bombesin. The labeling conditions were explored, but no biological data are available on the bioconjugates as of the time of this writing.^53–55^

### Click chemistry applied to chelator-biomolecule conjugation

Click chemistry, as first reported by K. Barry Sharpless in 2001,^56^ has been embraced by the PET radiopharmaceutical field as a new way to incorporate radionuclides via prosthetic groups or chelators.^57^ The most commonly used approach is the Huisgen 1,3-dipolar cycloaddition, also called copper-catalyzed alkyneazide cycloaddition (CuAAC). This reaction benefits from high chemoselectivity, fast reaction kinetics, and high stereoselectivity and results in high purity products in high yield in a wide spectrum of solvents. A series of click-to-chelate metal chelators were developed on the basis of Huisgen 1,3-dipolar cycloaddition chemistry.^58^ However, applications of CuAAC click chemistry for copper chelators is technically challenging because it is difficult to remove the copper from the chelator under conditions that can be tolerated by the bioconjugate, especially for sensitive biomolecules such as antibodies or peptides. Knör *et al*. synthesized ethynylphenyl-DOTA (**26**) and 4-acetylphenyl-DOTA (**27**) for chemoselective conjugation with azido-functionalized and aminooxy-functionalized Tyr^3^-octreotate, respectively.^59^ They removed the chelated copper from the final product through precipitation with sodium sulfide. Lebedev *et al*. applied a similar strategy on propargyl derivatized *tert*-butyl protected DOTA (**28**) and a CB-cyclam-based chelator (**29**).^60^ It is noteworthy that the newly formed triazole functioned as a new chelating unit that resulted in the ability to label ^64^Cu to the cross-bridged chelator under milder labeling conditions than CB-TE2A.

Strain-promoted copper-free click chemistry is especially attractive for conjugating copper chelators in that there is no need to remove the catalyst from the clicked product. Baumhover *et al*. functionalized DOTA and NOTA with a monofluorocyclooctyne (**30** and **31**) and conjugated them with an azide-modified peptide.^61^ Chen *et al*. modified diamsar with commercially available DBCO-PEG4-NHS ester and conjugated it (**32**) with an azido-modified RGD peptide,^62^ and the PET imaging and biodistribution results showed high specific uptake in the tumor with relatively low uptake in other tissues.

### *In vitro* evaluation of copper chelators

Factors that influence the *in vivo* stability of copper chelators have been evaluated.^63^ Through the pharmacokinetic evaluation of three cyclen-based and three cyclam-based chelators, it was concluded that negatively charged and neutral complexes have superior clearance profiles compared with positively charged complexes. More recently, the *in vivo* stability of copper complexes with CB-TE2A, CB-TE2P, CB-TE1A1P, NOTA, and diamsar were evaluated.^46^ For more precise measurement on *in vivo* stability, the Anderson group has performed metabolism studies to determine the extent of transchelation of ^64^Cu from radiometal chelates^37^ and from radiometalchelate-peptide conjugates.^29^ However, it is not always feasible or practical to apply animal biodistribution and/or metabolism studies for the evaluation of all the possible chelators. *In vitro* or *in silico* assays that mimic the *in vivo* environment that influences the copper dissociation are needed to predict the *in vivo* stability of the chelator conjugates. Traditionally, acid-promoted copper decomplexation was used as a measure of kinetic stability of the copper-chelator complex.^64^ This proved to give results that were somewhat in accordance with *in vivo* stability of the chelates than the dissociation constants, which measure thermodynamic stability. Cyclic voltammetry has also been applied to measure the reduction potentials of copper-chelator complexes,^64^ taking into consideration that the loss of copper *in vivo* may be attributable to the reduction of Cu(II) to Cu(I) with subsequent Cu(I) dissociation from the chelator.^64^ Packard and colleagues reported a simple method for measuring the copper exchange rate *in vitro*,^65^ where excess cold copper was used to exchange with chelated ^64^Cu at different pHs and temperatures. The results from three chelators were consistent with the trend of *in vivo* stability.

However, a larger pool of chelators is needed to validate further to support this copper exchange assay. It is also questionable whether a highly kinetically stable Cu(II) chelate will provide meaningful data in this assay.

### Nonchelating nanocarriers

Besides chelating copper, nonchelating methods to complex copper have been reported, such as the use nanoparticles to enclose copper. Petersen *et al*. patented this novel ‘labeling’ technique, which uses liposomes to entrap ^64^Cu without the use of ionophores.^66^ Tumors were imaged through the enhanced permeation and retention mechanism. Copper has also been incorporated into metal matrices. Using this strategy, Zhou *et al*. synthesized a ^64^Cu-doped CuS nanoparticle agent for PET imaging and photothermal ablation of tumor through the enhanced permeation and retention effect.^67^

### Factors involved in the specific activity of copper radionuclides

A critical factor in labeling chelator-biomolecule conjugates with copper radionuclides is the specific activity (amount of radioactivity per unit mass), which has been reported to be in the range of 94 to 310 mCi/μg for ^64^Cu.^14^ The effective specific activity is the ability to label a specific Cu(II) chelator at a particular temperature, pH, and for a specific amount of time. Therefore, the highest achievable specific activity is limited by the chelator used and the purity of the produced ^64^Cu, which includes the presence of other transition metals such as Ni, Zn, Fe, and Co. Zeng and Anderson recently reported a novel way to determine the specific activity of copper radionuclides using liquid chromatography–mass spectrometry analysis.^68^

This study demonstrates the importance of the specificity of chelators for Cu(II) compared with other transition metals, as well as their ability to efficiently chelate Cu(II) at a low concentration and form complexes with good kinetic and thermodynamic stability.

## Conclusion

Recent studies comparing peptide, peptidomimetic, or antibody bioconjugates with different chelators all point to the conclusion that is the choice of chelator is critical, as it influences the radiolabeling of the bioconjugate, targeting and pharmacokinetics.^36,69,70^ As new copper chelators are developed, we will have more choices that allow a better match with specific nanoparticles, antibodies, antibody fragments, peptides, peptidomimetics, or small molecules. DOTA is still the most widely used chelator, even though it is well known that ^64^Cu-DOTA complex has only modest *in vivo* stability, likely because of its commercial availability, Food and Drug Administration approval, and mild labeling conditions.^71^ Overall, cross-bridged cyclam derivatives are still the chelators with the best kinetic stability *in vivo*. The modifications of either the cross-bridge or the pendant arms improved the labeling condition without compromising the *in vivo* stability. With great efforts in copper chelator development, there has been less work done so far translating them toward clinical use. With comparable efforts in the translational direction, we would expect more impact of copper-64 in the field of nuclear medicine.

## Figures and Tables

**Table 1:** Production and physical properties of copper radionuclides relevant to nuclear medicine ^12,72^

Isotope	*t*_1/2_ (h)	method of production	decay mode	*E*_*β*^+/−^_ (keV)
^60^Cu	0.4	cyclotron, ^60^Ni(p,n)^60^Cu	β^+^ (93%); EC (7%)	3920, 3000; 2000
^61^Cu	3.3	cyclotron, ^61^Ni(p,n)^61^Cu	β^+^ (62%); EC (38%)	1220, 1150; 940, 560
^62^Cu	0.16	cyclotron, ^62^Zn/^62^Cu generator	β^+^ (98%); EC (2%)	2910
^64^Cu	12.7	cyclotron, ^64^Ni(p,n)^64^Cu	β^+^ (18%); EC (44%) β^−^(38%)	653 579
^67^Cu	62.01	reactor, ^67^Zn(n, p)	β^−^ (100%)	577, 484, 395
